# Amplification of GTP-cyclohydrolase 1 gene in *Plasmodium falciparum* isolates with the quadruple mutant of dihydrofolate reductase and dihydropteroate synthase genes in Ghana

**DOI:** 10.1371/journal.pone.0204871

**Published:** 2018-09-28

**Authors:** Musah Osei, Felix Ansah, Sena A. Matrevi, Kwaku P. Asante, Gordon A. Awandare, Neils B. Quashie, Nancy O. Duah

**Affiliations:** 1 West African Centre for Cell Biology of Infectious Pathogens (WACCBIP), Department of Biochemistry, Cell and Molecular Biology, School of Biological Sciences, College of Basic and Applied Sciences, University of Ghana, Legon, Accra, Ghana; 2 Kintampo Health Research Centre, Kintampo, Ghana; 3 Department of Epidemiology, Noguchi Memorial Institute for Medical Research, College of Health Sciences, University of Ghana, Legon, Accra, Ghana; 4 Centre for Tropical Clinical Pharmacology and Therapeutics, School of Medicine and Dentistry, College of Health Sciences, University of Ghana, Legon, Accra, Ghana; Instituto Rene Rachou, BRAZIL

## Abstract

Sulfadoxine-pyrimethamine (SP) is used as malaria chemoprophylaxis for pregnant women and children in Ghana. *Plasmodium falciparum* resistance to SP is linked to mutations in the dihydropteroate synthase gene (*pfdhps*), dihydrofolate reductase gene *(pfdhfr)* and amplification of GTP cyclohydrolase 1 (*pfgch1*) gene. The *pfgch1* duplication is associated with *pfdhfr* L164, a crucial mutant for high level pyrimethamine resistance which is rare in Ghana. The presence of amplified *pfgch1* in Ghanaian isolates could be an indicator of the evolution of the L164 mutant. This study therefore determined the *pfgch1* copy number variations and SP resistance mutations in clinical isolates from Ghana. One hundred and ninety-two (192) blood samples collected from children aged ≤14 years with uncomplicated malaria in 2013–14 and 2015–16 were used. Quantitative real-time polymerase chain reaction (qRT-PCR) was used to detect the *pfgch1* copy number and nested PCR-Sanger sequencing used to detect mutations in *pfdhps* and *pfdhfr* genes. Twelve parasites (6.3%) harbored double copies of the *pfgch1 g*ene out of the 192 samples. Of the 12, 75% had the *pfdhfr* I51-R59-N108, 92% had the *pfdhps* G437 mutant, 8% had the *pfdhps* E540 and 67% had the SP resistance haplotype IRNG. No L164 was detected in samples with amplified *pfgch1*. The rare T108 mutant associated with cycloguanil resistance showed predominance (60%) over N108 in the 2015–16 isolates. The observation of parasites with increased copy number of *pfgch1* gene is indicative of the future evolution of the rare quadruple *pfdhfr* mutant, I51-R59-N108-L164, in Ghanaian parasites. Mutant *pfdhps* isolates also had increased *gch1* copy number suggestive that it may also facilitate sulphadoxine resistance. The selection of parasites with *pfgch1* gene amplification will enhance the sustenance and persistence of parasites with SP resistance in the country. Policy makers need to begin the search for a replacement chemoprophylaxis drug for malaria vulnerable groups in Ghana.

## Introduction

Malaria is still a devastating disease in sub-Saharan Africa especially in children under the age of 5 years, pregnant women and non-immune travelers to the region. Chemoprophylaxis against the disease in these vulnerable groups involve the use of antifolate drugs, i) sulphadoxine-pyrimethamine (SP) is used for intermittent preventive treatment in pregnant women (IPTp) and seasonal malaria chemoprophylaxis (SMC) for children under 5 in seasonal malaria transmission areas; ii) atovaquone-proguanil (Malarone) is used by non-immune travelers to the malarious areas [1]. These antimalarials kill parasites by targeting enzymes in its *de novo* folate pathway such as the dihydrofolate reductase (DHFR) and dihydropteroate synthase (DHPS). The inhibition of these enzymes in the pathway, limits the availability of folate derivatives that serve as one-carbon carriers during nucleotide biosynthesis and amino acid metabolism in the parasite. Mutations in these genes therefore decrease the binding affinity of the drugs to the targeted enzymes because of the changes in the conformity of the protein [2].

*P*. *falciparum* resistance to SP has been linked to single nucleotide polymorphisms (SNPs) in the *pfdhfr* (pyrimethamine resistance) and *pfdhps* (sulphadoxine resistance) genes. The SNPs in the *pfdhfr* gene are Asn51Ile (N51I), Cys59Arg (C59R), Ser108Asn (S108N), and Ile164Leu (I164L) whilst those of the *pfdhps* gene are Ser436Ala/Phe (S436A/F), Ala437Gly (A437G), Lys540Glu (K540E), Ala581Gly (A581G), and Ala613Tyr (A613S/T) [3–7]. The S108N mutation is the *pfdhfr* core mutation which predicts a 10 fold increase in parasite resistance to pryrimethamine whilst the core mutations for sulphadoxine resistance are *pfdhps* mutationsA437G and G540E [8]. The resistance level of parasitesis linked to the number of point mutations or haplotypes of the 2 genes and therefore, multiple mutations are responsible for high levels of SP resistance [9]. There is a synergistic effect in conferring resistance for SP with the quintuple mutant of *pfdhfr* and *pfdhps*, haplotype I51-R59-N108-G437-E540 [10]. Reports of increasing prevalence of these mutations have been reported in African malaria endemic areas including Ghana [8,11–13].

The efficacy of SP for malaria prevention might have reduced drastically because of reports from surveillance studies of circulating parasites with resistance haplotypes [14]. This observed increase in parasites with resistance haplotypes is due to selection by drug pressure from the use of SP for IPTp and SMC as well as the use of antifolate containing antibiotics in the country. Genomic analyses of the malaria parasites showed copy number variations (CNV) of GTP cyclohydrolase I gene (*pfgch1*) which encodes the first and the rate-limiting enzyme of the *de novo* folate biosynthesis. Increased copy number of *gch1* has been linked to SP resistance in Southeast Asia (SEA) [15,16]. Multiple copies of *pfgch1* have been shown to have a direct association with point mutations in *pfdhfr* but not *pfdhps* in SEA, which was confirmed in a separate study using genetic manipulations of parasite lines[17,18]. The increased copy number of *pfgch1* were detected in a study looking at geographically distinct parasites with known drug resistance profiles [18]. In Africa, a recent study using Malawian parasites reported a novel *pfgch1* promoter duplication in parasites with quintuple mutations (I51-R59-N108-G437-E540) which differs from the whole gene duplication found in Southeast Asia [19].

The drug-resistant mutations in the two genes, though advantageous under SP pressure, they render the parasite less fit due to the changes at the enzymes active sites [20–22]. It was therefore suspected that multiple copies of *gch1* could compensate for the putatively fitness-reducing mutations in *pfdhfr* and *pfdhps* by providing higher concentrations of downstream substrates in its folate-biosynthetic pathway [23]. This phenomenon may also directly increase the level of SP resistance in the parasite[17]. A joint analysis of *gch1* CNVs, *pfdhfr* and *pfdhps* SNPs may therefore shed more light on the molecular mechanisms of SP resistance and how these resistant isolates are maintained in circulation in malaria endemic areas like Ghana.

## Materials and methods

### Study design and sites

**Fig 1:**
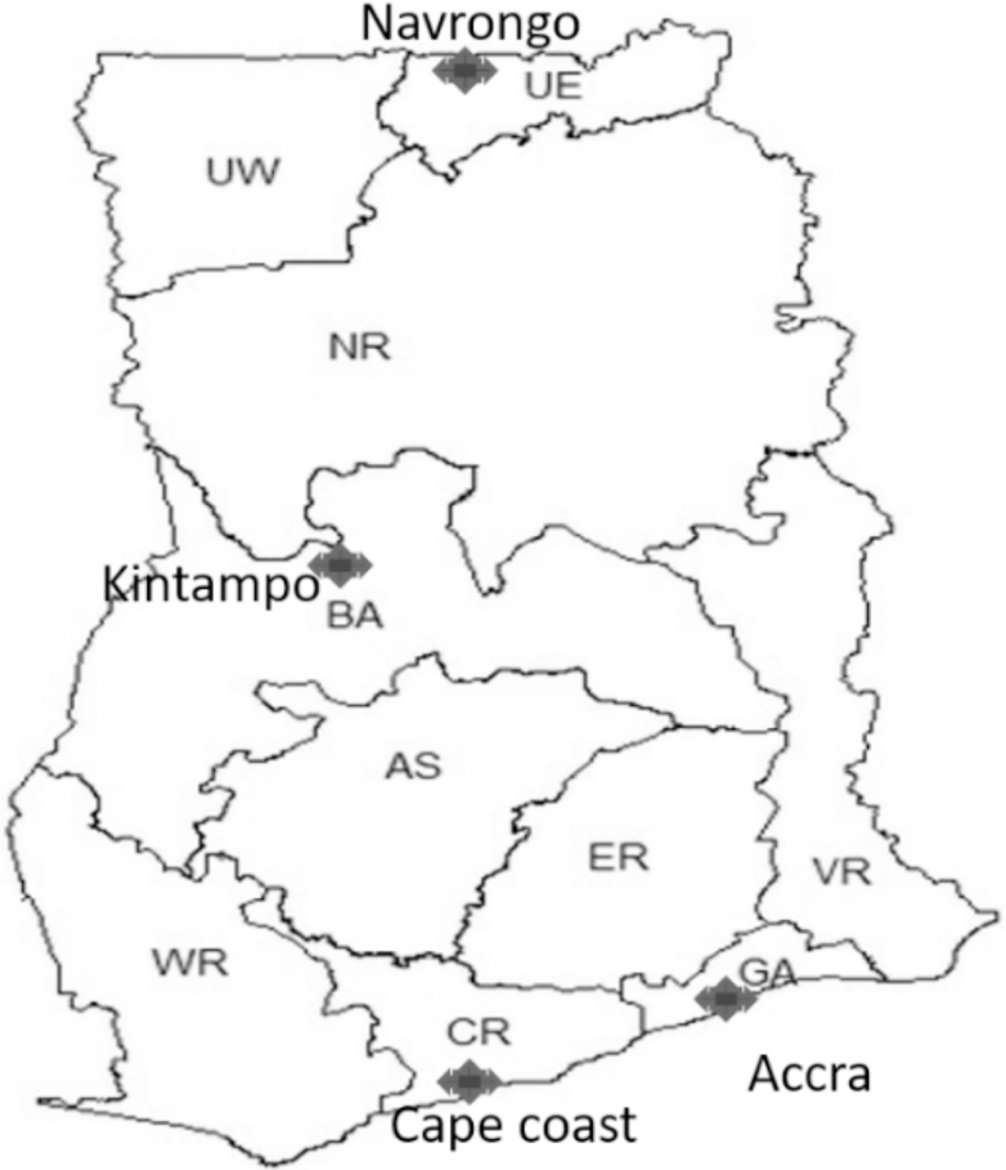
Map of Ghana showing the four study sites. Sites shown by black arrows.

Filter paper blood blots were prepared from whole blood samples collected from children aged 6 months to 14 years with uncomplicated malaria in 2013–2016 from four sentinel sites for monitoring antimalarial drug efficacy, Accra, Cape Coast, Kintampo and Navrongo (Fig 1). These sites are located in the three different ecological zones in Ghana with different malaria transmission intensities. These children were enrolled after obtaining informed consent from their parents or guardians to participate in the studies.

### Ethical consideration

Ethical approval was obtained from the ethics committees and Institutional Review Boards (IRB) of the Ghana health service, the Noguchi Memorial Institute of Medical Research, the Kintampo Health Research Centre and the Navrongo Health Research Centre prior to the conduct of these studies. The proposals for ethical clearance stated the categorical use of the samples for future molecular analysis. A written informed consent was used and the IRB approved the consent procedure.

### Molecular analysis

#### DNA extraction and nPCR amplification of genes

Genomic DNA was extracted from 202 dried blood blot filter paper samples using the QIAamp DNA Blood Mini kit following the manufacturer’s protocol. The nested polymerase chain reaction (nPCR) was used to amplify the target region within the *pfdhfr* and *pfdhps* genes with fragment sizes of 616bp and 647bp, respectively following protocol [24,25] with minor modifications. A 25μl reaction mix contained 1U High Fidelity *Taq* polymerase (Roche, UK), 0.2mM dNTP mix, 1.5mM MgCl_2_ and 0.25μM of each primer and 2μl DNA template for the primary PCR. For the secondary PCR, the same reaction concentrations were used as in the outer PCR but in a final volume of 50 μl. The *pfdhfr* primers were F1: 5’-TCC TTT TTA TGA TGG AAC AAG-3' and R1: 5’-AGT ATA TAC ATC GCT AAC AGA-3'; F2: 5’- TTT ATG ATG GAA CAA GTC TGC -3' and R2: 5’-ACT CAT TTT CAT TTA TTT CTG G-3' and those for *pfdhps* were F1: 5’-AAC CTA AAC GTG CTG TTC AA-3' and R1: 5’-AAT TGT GTG ATT TGT CCA CAA-3'; F2: 5’-ATG ATA AAT GAA GGT GCT AG-3' and R2: 5’-TCA TTT TGT TGT TCA TCA TGT-3'. The cycling conditions for both genes were for the primary reaction, an initial denaturation of 94°C for 5 mins followed by 35 cycles of 95°C for 30secs, 50°C for 30secs and 68°C for 1min and final extension of 68°C for 5 mins and the secondary reactions had an annealing temperature of 52°C and 30 cycles. PCR products were analyzed on a 2% agarose gel with ethidium bromide to determine the specificity and success of the PCR for sequencing.

#### Sequencing of *pfdhfr* and *pfdhps* genes

Sanger sequencing of both *dhfr* and *dhps* fragments were performed at Macrogen (Denmark). One hundred and ninety two of the 202 samples were successfully sequenced. Samples with multiple peaks at any of the codons genotyped were trimmed and only high-quality peaks were called and included in the analysis. The sequenced results in the ABI file format were analysed using Qiagen CLC Main Workbench analysis software (version 7.8.1) and the Codon Code Aligner software (version 7.0.1). Detection of SNPs and haplotypes were done by aligning the sample sequences with the reference 3D7 (Wild-type) sequences retrieved from www.Plasmodb.org.

#### Quantitative real-time PCR to determine copy number variation of *pfgch1*

QRT-PCR was carried out on all the 202 DNA samples to determine copy numbers using the SYBR-Green method by Heinberg et al [17] with minor modifications. All reactions were performed at primer concentrations of 0.5 μM, 1X PerfeCTa SYBR Green Supermix (Quantabio) and 2μl of DNA template. Each sample was run in triplicate using the QuantStudio5 (Applied Biosystems) real-time PCR machine. The *gch1* primers were F: 5'-ATG AAA CAC ATA ATA TGG AAG AAA AA-3 and R: 5'-TCC TTT TCA TCT ATC ACA ACA AGG-3'; seryl-tRNA synthetase primers were F: 5'-AAG TAG CAG GTC ATC GTG GTT-3' and R: 5'-TTC GGC ACA TTC TTC CAT AA-3'). The cycling conditions were an initial 50°C for 2 mins and 95°C for 1 min followed by 40 cycles of 95°C for 30 secs, 54°C for 40 secs and 60°C for 1 min and a melt curve stage of 95°C for 15 secs, 60°C for 1 min and 95°C for 1 sec. The seryl-tRNA synthetase was used as the endogenous control. Reference samples (Dd2 and 3D7) with known *gch1* copy numbers and a non-template negative controls were included in each run. Samples with amplified *gch1* copy number were repeated for certainty. The melting curve analysis was performed for each run and experiments with non-specific products, Ct > 32 or standard deviation (SD) ≥ 0.5 were repeated. The delta delta Ct formula (2^-ΔΔCt^) was used to estimate the relative copy numbers.

#### Determination of *pfgch1* gene expression levels

Clinical isolates harboring different copies of the *pfgch1* gene and laboratory strain Dd2 (control) were thawed and put into continuous culture following the protocol by Trager and Jensen [26]. Briefly, the thawed parasites were suspended in a complete parasite medium (CPM) at 5% hematocrit using un-infected human group O+ erythrocytes. At a parasitaemia of 2 to 5%, the parasites were synchronized using sorbitol and put back into culture until they developed into late trophozoites, a stage where the *pfgch1* has been shown to be highly expressed [27]. The RNA was isolated using AllPrep DNA/RNA Mini kit protocol by Qiagen (Qiagen, Germany) and the RNA samples treated with DNase to remove all residue DNA. The cDNA synthesis was then carried out with a control reaction (without a reverse transcriptase) using the Superscript III first-strand protocol following manufacturer’s instructions (ThermoFisher Scientific, USA). The cDNA synthesis reaction condition includes 25°C for 10 min, 50°C for 50 min and 85°C for 5 min. Following the cDNA synthesis, 1 μl of RNase H was added to each reaction, mixed and incubated at 37°C for 20 min and 95°C for 10 min to remove RNA residues. QRT-PCR was then carried out on the cDNA with the same reaction conditions as described for the copy number determination and the Ct values converted to expression levels using the 2^-ΔΔCt^ formula [28].

### Statistical analysis

Data were analysed using SPSS software (version 20) and GraphPad Prism version 6. Descriptive analyses for mutations were performed. Chi-square and multiple comparison tests were used to compare the difference between groups. All tests were considered statistically significant with P<0.005.

## Results

Conventional nested PCR (nPCR) and Sanger sequencing were successfully performed on 192 samples for the detection of SNPs in the *pfdhfr* and *pfdhps* genes. Quantitative real-time PCR (qRT-PCR) was performed on the same number of samples for the copy number variation in *gch1* gene. Of the 192 samples, 100 were collected in 2013–14 and 92 in 2015–16. The sites for the 2013–14 samples were Accra (37), Kintampo (39) and Navrongo (24) and those of 2015–16 were Cape-Coast (47) and Navrongo (45).

### Prevalence of mutations in *pfdhfr and pfdhps*

The sequence analysis of the SNPs in the *pfdhfr* gene revealed that the prevalence of I51, R59, N108 and T108 in 2013–14 to be 91%, 92%, 95% and 7% respectively. The prevalence of these mutation in 2015–16 was 82.65, 80.6%, 47.8% and 59.9% respectively for I51, R59, N108 and T108. Only one sample had the wildtype S108 in the 2013–14 samples but none had the wildtype in the 2015–16 samples. Three samples had both N108 and T108 in the 2013–2014 and 7 had both mutations in the 2015–16 samples. An increasing trend for T108 was observed which seems to be taking over N108 prevalence especially in Navrongo. No mutations were detected at codons 50 and 164 of the *pfdhfr* gene. The distribution of the mutations per site is shown in Table 1.

**Table 1:** Proportions of samples per site with *pfdhfr* and *pfdhps* mutations.

Sites		*pfdhfr* mutations				*pfdhps* mutations				
	n	I51	R59	N108	T108	A436	G437	E540	G581	S613
**2013–14**										
Accra	37	86.5	86.5	97.3	0	24.3	59.5	0	5.4	8.1
Kintampo	39	94.9	92.39	92.3	7.7	28.2	66.7	2.6	0	5.1
Navrongo	24	91.7	100	95.8	16.7	37.5	75.0	0	0	0
**2015–16**										
Cape-Coast	47	80.9	76.6	42.6	57.5	27.7	78.7	0	2.1	10.6
Navrongo	45	84.4	84.4	53.3	62.2	44.4	82.2	0	2.2	13.3

**Fig 2:**
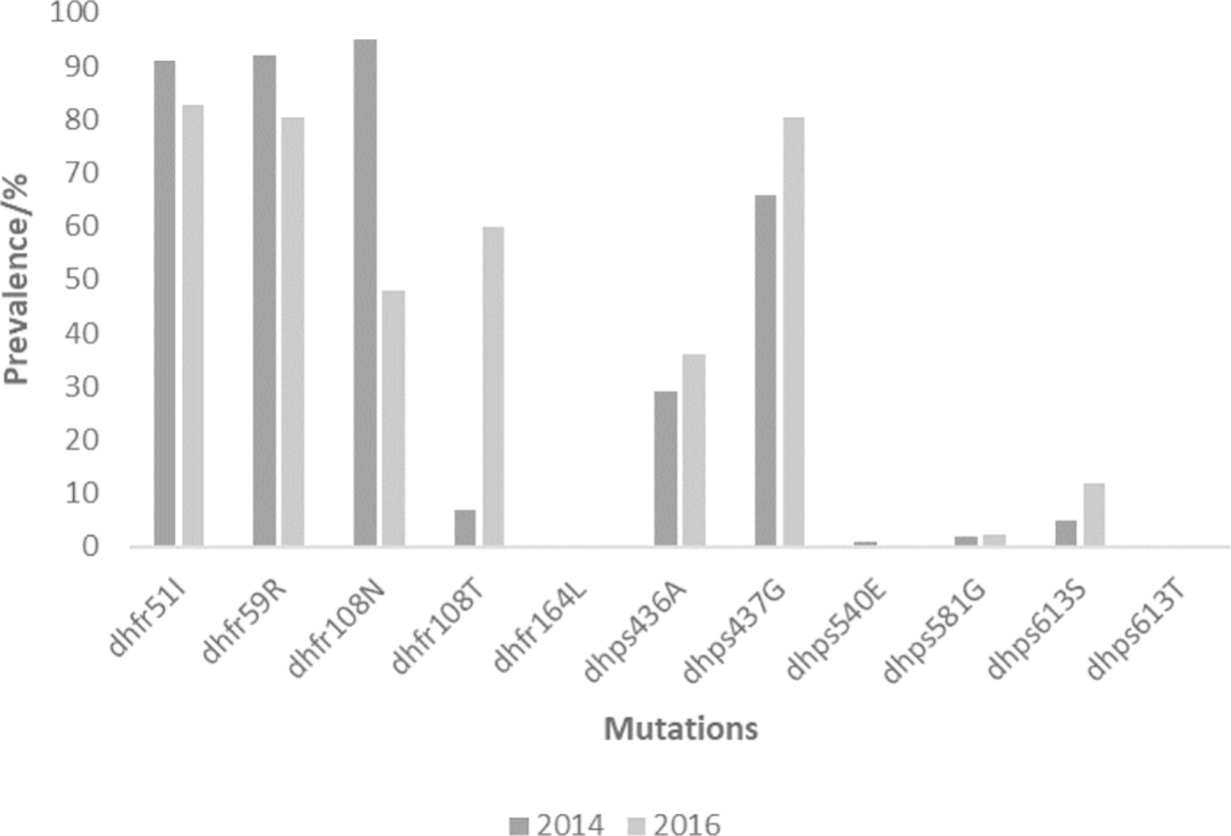
The prevalence of the *pfdhfr* and *pfdhps* mutations for the two time points in Ghana.

For the *pfdhps* mutations the prevalence for 2013–14 were, 29.0%, 66.0%, 1.0%, 2.0% and 5.0% for A436, G437, E540, G581, S613 respectively. The prevalence for 2015–16 for A436, G437, E540, G581, S613 were 35.9%, 80.4%, 0%. 2.2% and 11.9% respectively. No T613 mutation was observed in all samples. The distribution of the *pfdhps* mutations is per site is also shown in Table 1. The overall prevalence of the *pfdhfr* and *pfdhps* mutations in the country for the two time points are shown in Fig 2.

### Prevalence of haplotypes of *pfdhfr* and *pfdhps*

**Fig 3:**
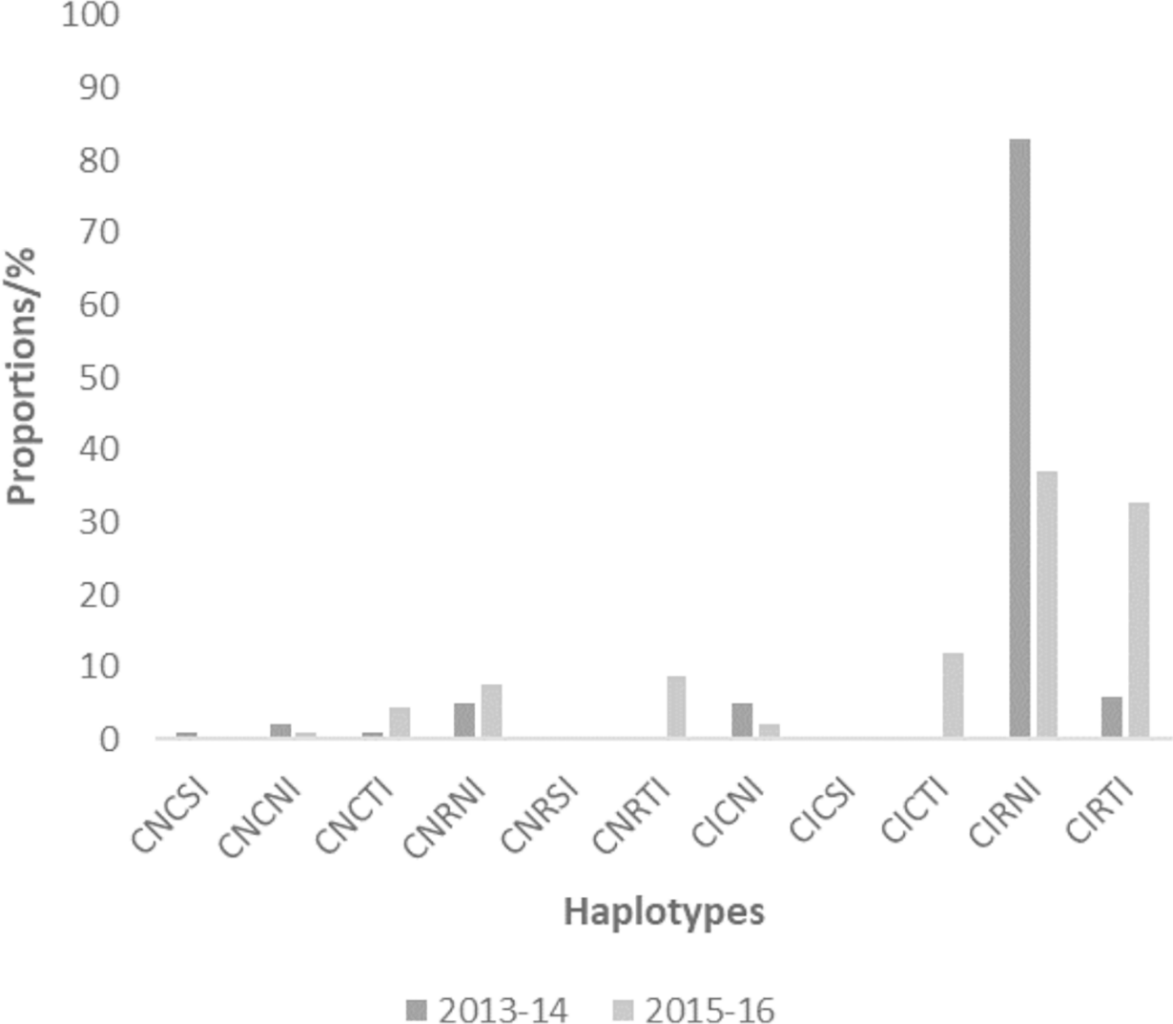
The proportion of isolates with *pfdhfr* haplotypes for the two time points in Ghana.

**Fig 4:**
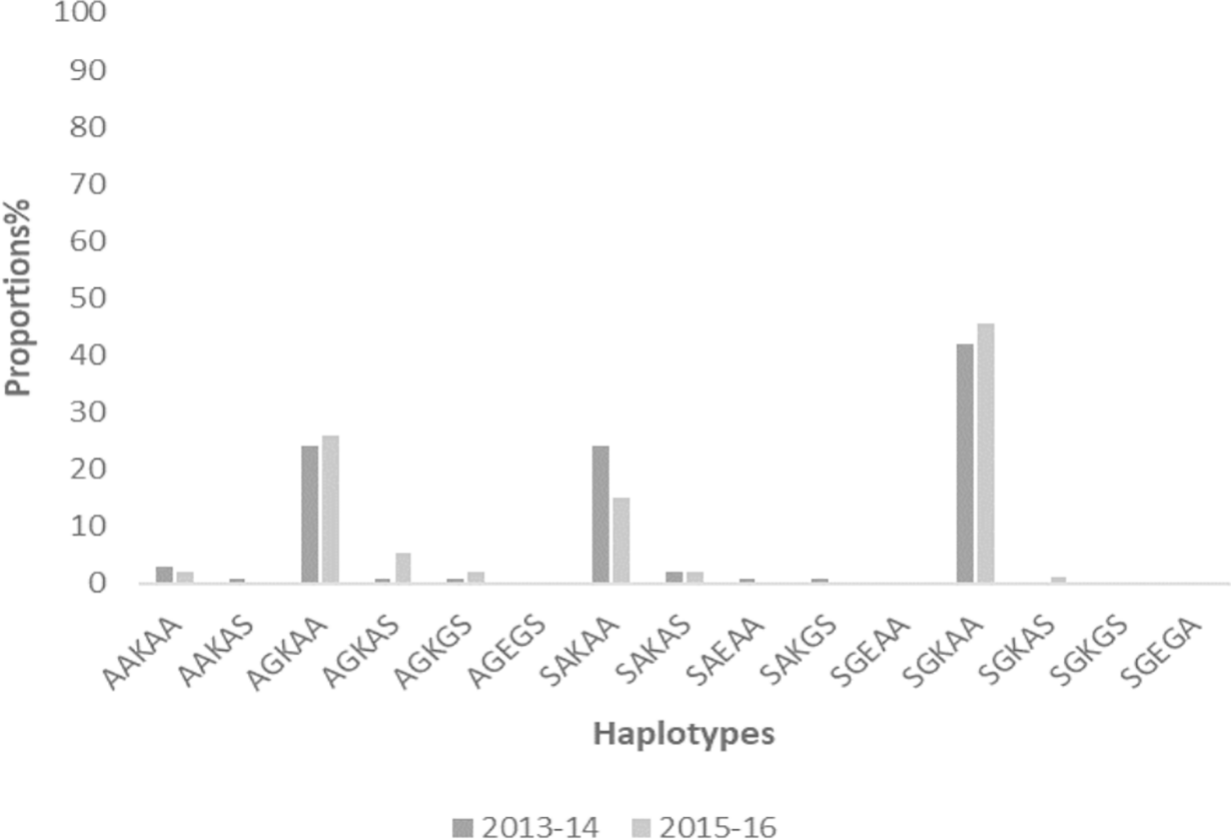
The proportion of isolates with *pfdhps* haplotypes for the two time points in Ghana.

**Fig 5:**
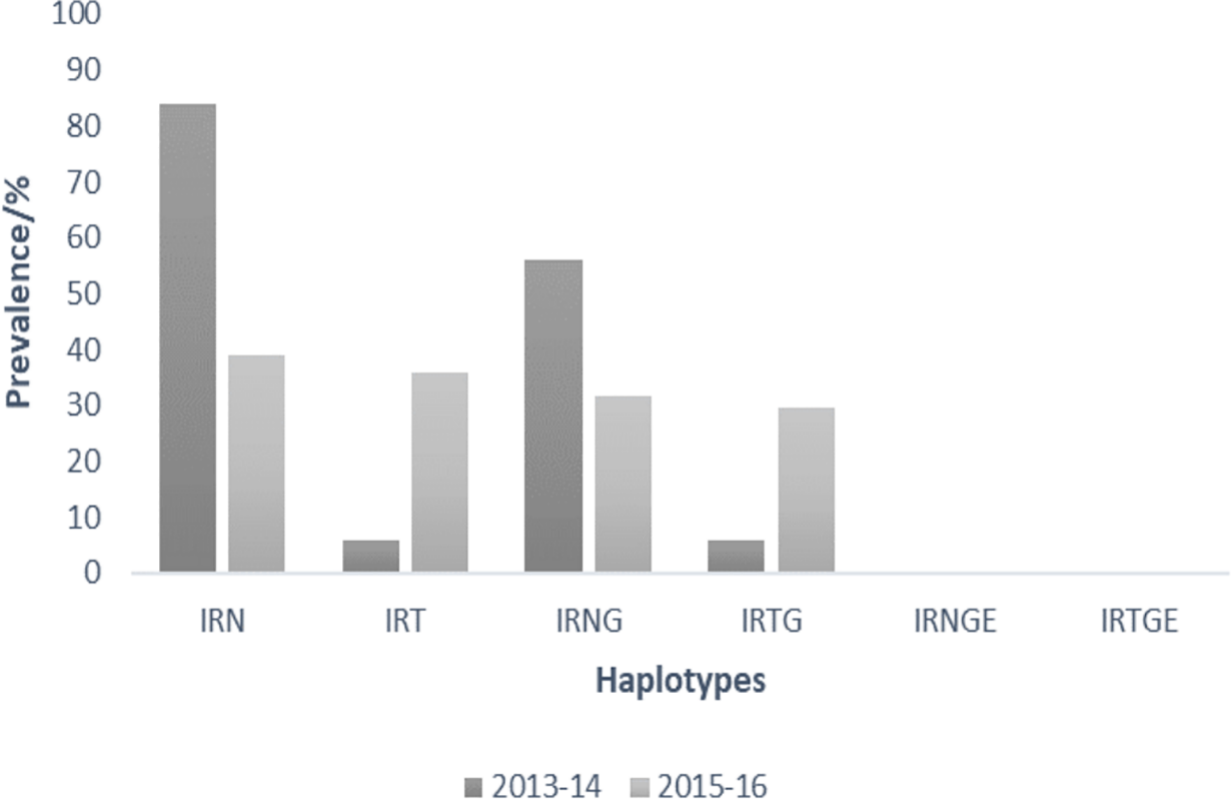
The prevalence of the SP and cycloguanil resistance haplotypes for the two time points in Ghana.

Proportions of samples from the two time points with haplotypes from *pfdhfr* codons 50-51-59-108-164 and *pfdhps* codons 436-437-540-581-613 are shown in Figs 3 and 4. The prevalence of mutant haplotypes associated with pyrimethamine resistance, *pfdhfr* triple mutants I51-R59-N108 (IRN) and I51-R59-T108 (IRT), as well as those for sulphadoxine resistance, *pfdhps* double mutant G437-E540 (GE), quadruple mutant IRNG and quintuple mutant IRNGE are shown in Fig 5. No quintuple mutant was observed in the samples for both 2013–14 and 2015–16 timepoints.

### Copy number variations of the *pfgch1* gene in *P*. *falciparum* isolates

**Fig 6:**
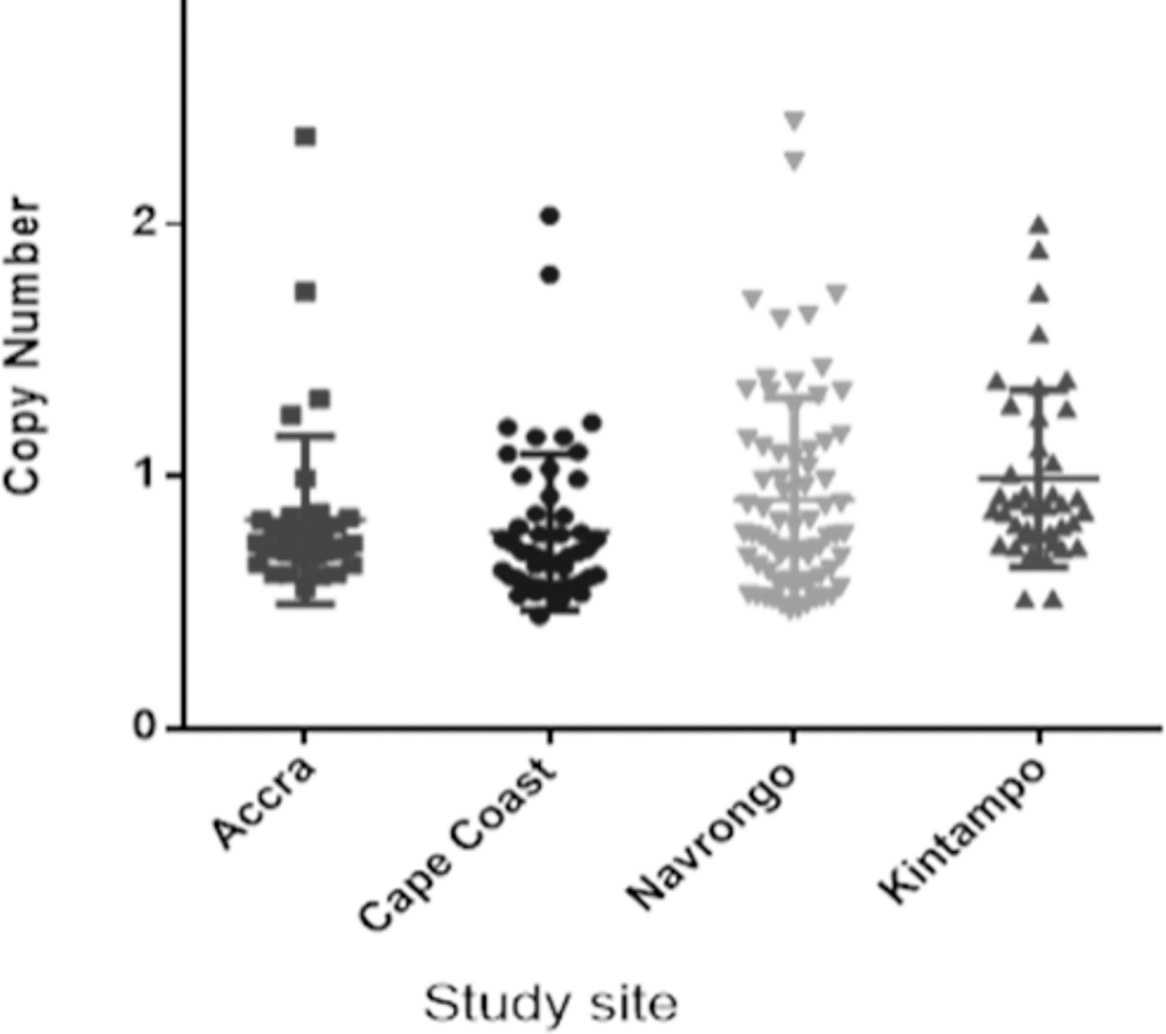
The scatter plot of estimated *pfgch1* copy number per site. Each dot represents the estimated copy number.

*Gch1* copy number (CN) was determined in 192 samples, of which 6.3% (12/192) had an estimated copy number of 2. The distribution of the estimated CN from the isolates is shown in Fig 6. The highest proportion with a CN of 2 was seen in the 75%, (9/12) of samples from 2013–14 of which Accra had 2, Kintampo had 4 and Navrongo had 3. Only 3 samples from 2015–16 had a CN of 2, 2 from Navrongo and 1 from Cape-Coast.

### Expression levels of *pfgch1* in field isolates

To ascertain the transcriptional importance of *gch1* gene dosage, the expression levels of DD2 strain and field isolates with differing *gch1* gene copies were determined. Isolates with a single copy had a mean relative expression of 0.44 as compared to 1.55 expression level amongst parasites with double *gch1* gene as shown in Table 2.

**Table 2:** The gch1 expression levels in field isolates with varying gene copy number.

Sample ID	*pfgch1* CN	*pfgch1* expression level
Dd2	2	1.454
EIMK526	2	1.589
EIMK481	2	1.602
EIMA226	1	0.582
EIMA237	1	0.30

### *pfdhfr* and *pfdhps* SNPs and haplotypes with increased *pfgch1* CN

The pf*dhfr* and *pfdhps* mutations and increased *gch1* copy number were analysed to assess their dependence. The SNPs with the increased *gch1* gene included the triple mutant IRN of the *pfdhfr* gene and the G437 the *pfdhps* gene. Of the 12 samples with increased copy number, 75% (9/12) had the triple mutant IRN, 92% (11/12) had G437 mutation and 67% (8/12) had both IRN and G437 and 8% (1/12) had the E540. Of the 111 samples with the quadruple mutant, 8% (9/111) had the increased copy number of *pfgch1* whilst the majority had single copy of the gene. No significant difference was observed between those with increased copy number and single copy for the quadruple mutants (p = 0.236).

## Discussion

In this study, we determined the presence of amplified *pfgch1* gene and the *pfdhfr* and *pfdhps* SNPs in *P*. *falciparum* clinical isolates from Ghana. This is important to clarify the underlying factors for the sustenance and persistence of the less fit SP resistant parasites in the population. Trajectory analysis of the *pfgch1* amplification has revealed its role in the fixation of *pfdhfr* mutants in the parasite population but with a minor influence in directing drug resistance [17,28] Indeed, for parasites with multiple *pfdhfr* mutations, duplication of the *pfgch1* gene provided fitness advantage rather than directly reducing pyrimethamine susceptibility. This study detected increased copy number of *pfgch1* gene in Ghanaian clinical isolates and observed that the copy number corresponds to the gene expression levels. The presence of the amplified gene did correlate not only with *pfdhfr* mutations as reported from other studies but also with *pfdhps* mutations in Ghanaian isolates. Since the quadruple mutant of *pfdhfr* with L164 which confers high level pyrimethamine resistance has been linked to the presence of increased *pfgch1* copy number [18,19], it could be indicative of a near future evolution of this rare mutation in the parasite population of Ghana. The consequence of this may render the prophylactic drug for the vulnerable groups ineffective.

Until recently, increased *pfgch1* copy number was seen only in isolates from the SEA region specifically Thailand [29]. CNV of this gene has been associated with pyrimethamine resistance in SEA and South Asia [16,18,19]. For the first time, a report from Ravenhall and others showed increased *pfgch1* promoter gene in African isolates from Malawi, Ghana, Guinea, Gambia and the Democratic Republic of Congo [19]. However, they observed a whole duplication of the gene in isolates from Thailand, Cambodia, Vietnam, Myanmar and the only African country was Ghana (5%) [19]. This current study observed the amplification of the whole gene in about 7% of the clinical isolates and was linked predominantly with the triple mutant IRN as observed in other studies [19,28]. Interestingly, the whole gene amplification also correlated with the presence of *pfdhps* G437 in this study just as the promoter amplification seen in the Ghanaian isolates from the other study. The IRNG which is the haplotype for SP resistance also seen in about 70% of the samples with amplified *pfgch1*. The role of *pfgch1* in compensating for the fitness cost may be a result of increased copies of the gene which could lead to high transcriptional and enzyme levels by many folds [18,30]. The copy number observed in this study was directly proportional to the transcriptional levels and as enzyme levels increase, consequently there will be an increase the downstream metabolites which will affect activities of the DHFR and DHPS enzymes. Thus, in the less fit parasites with SP-resistant *pfdhfr* and *pfdhps* haplotypes such an adaptation compensates for their fitness cost [28].

The *pfdhfr* mutation at codon 108 is from serine to asparagine or threonine (S108N/T). It is worth mentioning that from studies conducted in the past on SP resistance markers in Ghana, the T108 has not been reported in Ghanaian isolates. This mutant associated with cycloguanil resistance [10,31,32] was prevalent in 7% of the isolates from 2013–14 and 60% of those collected in 2015–16. In all, 7 samples had both N108 and T108. It was observed that either the sample may have one or the other, however majority of the samples in the 2015–16 group had the T108. In addition, 2 samples with IRT had an increased *pfgch1* gene. Cycloguanil is a metabolite of proguanil which is in combination with atovaquone as malarone. Malarone is used by non-immune travelers to malaria endemic areas for prophylaxis, as such with this observation, amendment in treatment policies is recommended.

The high transmission areas of Navrongo and Kintampo seemed to have higher proportions of parasites with double *pfgch1* 50% and 33% respectively compared to the lower transmission sites, Accra and Cape-Coast with one sample each. The role of transmission and the evolution of mutations in parasites is quite clear especially for the *pfcrt*, *pfmdr1*,*pfdhfr* and *pfdhps* genes. The *pfgch1* copy number evolution is as a result of an adaptation to drug pressure [18,23], therefore the percentage prevalence observed in this study indicate a high use of antifolate drug in the country. It must be emphasized that although the use of SP in Ghana is restricted to pregnant women and children as prophylaxis, most chemical and pharmacy shops still stock and sell the drug to individuals outside these groups [33]. Another reason for the relatively high drug pressure may be the use of antifolate-containing antibiotics prescribed in the country.

After the change of antimalarial drug treatment policy in Ghana in 2005, SP had been used as an IPTp and there have been reports of increased SP resistance markers over the years[8,24]. Studies by Mockenhaupt *et al*. in 2005 reported the presence of 47% of *dhfr* triple and 0.8% of quintuple mutants [34]. The above frequencies are higher amongst parasites collected in 2007/2008 by Alam *et al*, where the triple *dhfr* and quintuple mutants were reported as 58.7% and 1.8% respectively [24]. Duah and colleagues also reported the prevalence of the triple *dhfr* and quintuple mutants in parasites collected in 2010 as 55% and 1.12% respectively[8]. A recent report by Abugri and colleagues have also affirmed an increasing trend in the triple and quadruple mutants for SP resistance [13]. The relatively high prevalence of the triple mutant IRN, 84% for 2013–14, declined to 39% due to the upsurge of the T108, such that IRT was 36%. This also affected the prevalence of the quadruple mutant IRNG which was 56% for 2013–14 but was 32% in 2015–16 and therefore the IRNTG was 29%. As such no quintuple mutants for SP resistance were observed in the current study although one sample from the 2013–14 group had the *pfdhps* E540 mutant but with a A437.

The increased prevalence of the known resistant markers in our study may be attributed to drug pressures, however the presence of the amplified *pfgch1* will exacerbate the existing problem and may help to maintain these resistant parasites and the evolution of new mutants. Drug pressure selecting these mutations is not only for SP use but also for other antifolates. A sulfonamide antifolate-containing antibiotic known as cotrimoxazole which is a combination of trimethoprim and sulfamethoxazole is often prescribed to treat pneumonia, bronchitis, infections of the urinary tract, ears and intestines, and as prophylaxis in HIV infected individuals. The administration of this drug in patients with bacterial and malaria co-infection may, therefore, lead to high drug pressure with a consequent selection for drug resistant parasites. The most critical scenario with this new parasite adaption is the ability of mutants to equally or favorably compete with the wild type in the absence or reduced SP drug pressure and thus cause the expansion and persistence of the resistant type. If this speculated mechanism holds, then it may jeopardize the use of SP drug and other antifolate-containing antibiotics in Ghana in the near future. It is interesting to note that there are still several reports of high cases of SP resistant parasites in areas with no or restricted use of the SP drug [35–37].

## Conclusion

The observation of that rare mutation (amplified *pfgch1*) in Ghanaian isolates indicates that there is an upcoming force to aid in the sustenance and persistence of parasites with SP resistance mutations. In addition, it may influence the evolution of new mutations to invoke a relatively higher level of resistance of SP in the Ghanaian parasite population. Therefore with the emergence of such a rare mutation in this geographical region, it is important that policy makers begin the search for a new chemoprophylactic drug to replace SP for the two most vulnerable groups, pregnant women and children in Ghana.

## Supporting information

S1 Table The list of all observed mutations in the *pfdhfr* and *pfdhps* genes as well as the estimated copy number of the *pfgch1* for all 192 samples analyzed. This excel sheet contains data supporting the findings in this study. A score of 1 depicts presence of a mutation whilst 0 is no mutation detected.
(XLSX)
